# Association between Neutrophil-Lymphocyte Ratio and Herpes Zoster Infection in 1688 Living Donor Liver Transplantation Recipients at a Large Single Center

**DOI:** 10.3390/biomedicines9080963

**Published:** 2021-08-05

**Authors:** Ji-Hoon Sim, Young-Jin Moon, Sung-Hoon Kim, Kyoung-Sun Kim, Ju-Seung Lee, Jun-Gol Song, Gyu-Sam Hwang

**Affiliations:** Department of Anesthesiology and Pain Medicine, Asan Medical Center, University of Ulsan College of Medicine, Seoul 05505, Korea; atlassjh@hanmail.net (J.-H.S.); yjmoon@amc.seoul.kr (Y.-J.M.); shkimans@gmail.com (S.-H.K.); kyoungsun.kim@amc.seoul.kr (K.-S.K.); christa416@naver.com (J.-S.L.); kshwang@amc.seoul.kr (G.-S.H.)

**Keywords:** herpes zoster, liver transplantation, living donor, neutrophil-lymphocyte ratio, postherpetic neuralgia

## Abstract

Liver transplantation (LT) is closely associated with decreased immune function, a contributor to herpes zoster (HZ). However, risk factors for HZ in living donor LT (LDLT) remain unknown. Neutrophil-lymphocyte ratio (NLR) and immune system function are reportedly correlated. This study investigated the association between NLR and HZ in 1688 patients who underwent LDLT between January 2010 and July 2020 and evaluated risk factors for HZ and postherpetic neuralgia (PHN). The predictive power of NLR was assessed through the concordance index and an integrated discrimination improvement (IDI) analysis. Of the total cohort, 138 (8.2%) had HZ. The incidence of HZ after LT was 11.2 per 1000 person-years and 0.1%, 1.3%, 2.9%, and 13.5% at 1, 3, 5, and 10 years, respectively. In the Cox regression analysis, preoperative NLR was significantly associated with HZ (adjusted hazard ratio [HR], 1.05; 95% confidence interval [CI], 1.02–1.09; *p* = 0.005) and PHN (HR, 1.08; 95% CI, 1.03–1.13; *p* = 0.001). Age, sex, mycophenolate mofetil use, and hepatitis B virus infection were risk factors for HZ versus age and sex for PHN. In the IDI analysis, NLR was discriminative for HZ and PHN (*p* = 0.020 and *p* = 0.047, respectively). Preoperative NLR might predict HZ and PHN in LDLT recipients.

## 1. Introduction

Herpes zoster (HZ) is a viral disease caused by varicella zoster virus (VZV) reactivation and has an estimated incidence of 1.2–4.8 per 1000 individuals in the general population [1]. HZ is strongly associated with decreased immune function [2,3]; thus, it occurs mainly in elderly individuals [4,5,6,7], patients infected with human immunodeficiency virus (HIV), and transplant recipients [8]. Previous studies reported that solid-organ transplant (SOT) recipients have a 2- to 10-fold higher risk of VZV reactivation than the general population [9,10,11]. In particular, liver transplantation (LT) recipients are at increased risk of developing HZ due to a chronic immunosuppressed status [12,13]. Several studies reported postoperative HZ in SOT recipients [8,9,10,11,12,13,14]. In one study, the overall incidence of HZ after transplantation was 8.6% (liver, 5.7%; renal, 7.4%; lung, 15.1%; and heart, 16.8%) [15]. Pergam and colleagues reported that LT patients generally require less immunosuppression because the liver is relatively resistant to rejection compared to other organs and that the incidence of HZ is slightly lower than that of lung and heart transplant patients [11]. Nevertheless, the incidence of HZ in LT patients (up to 12%) is higher than that of the general patient group [12,13,14].

Several recent reports stated that neutrophil-lymphocyte ratio (NLR) is associated with immune system function and the inflammatory response [16]. NLR is calculated as the ratio of the number of neutrophils and lymphocytes measured in the peripheral blood and reflects a patient’s inflammatory condition (neutrophil count) and adaptive immunity (lymphocyte count). NLR is a simple and objective biomarker of patient survival and surgical prognosis in various diseases, such as major cardiac disease and several cancers [17,18].

However, no studies to date have examined the association between preoperative NLR and HZ. Therefore, in this study, we evaluated the association between preoperative NLR and HZ development in patients who underwent living donor LT (LDLT). In addition, we assessed the association between preoperative NLR and postherpetic neuralgia (PHN) development, an advanced complication of HZ.

## 2. Materials and Methods

### 2.1. Study Design and Patients

This study was approved by the institutional review board of Asan Medical Center (protocol number: 2021-0526), which waived the requirement for written informed consent due to its retrospective design. We reviewed the data from all patients who underwent LDLT for end-stage liver disease between January 2010 and July 2020. Adult patients aged ≥18 years were included in the study.

**Figure 1:**
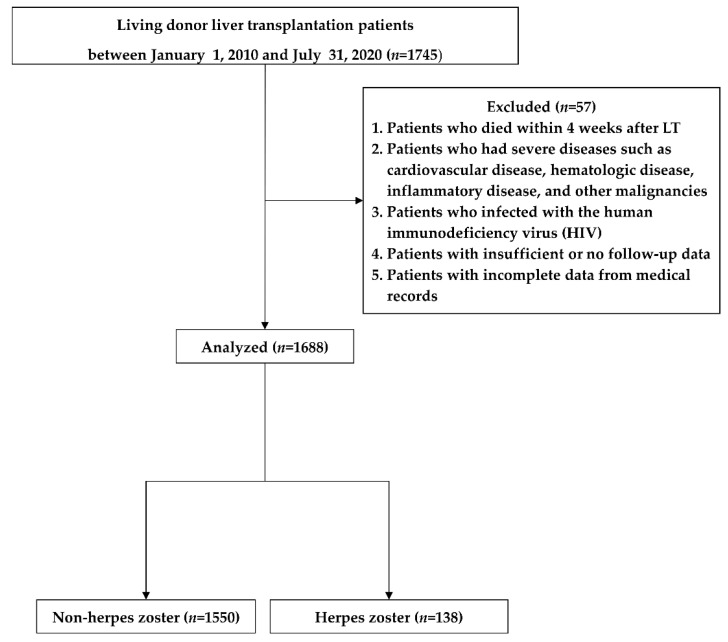
Flow chart of the study.

The exclusion criteria were as follows: (1) death within 4 weeks after LT; (2) presence of severe diseases such as hematologic disease, inflammatory disease, and other malignancies; (3) HIV infection; (4) insufficient or no follow-up data; and (5) incomplete medical record data. Based on these criteria, a total of 1688 patients were enrolled and divided into HZ (*n* = 138) and non-HZ (*n* = 1550) groups (Figure 1).

### 2.2. Clinical Data Collection and Outcome Assessments

Demographic data and perioperative variables were collected from our institution’s medical record system. Demographic data included age, sex, body mass index (BMI), diabetes mellitus (DM), hypertension (HTN), coronary artery disease (CAD), Model for End-stage Liver Disease (MELD) score, and Child–Pugh classification. Data on ABO incompatibility and retransplantation were also collected. Etiology-related variables included hepatitis B virus (HBV) infection, hepatitis C virus (HCV) infection, alcoholic liver cirrhosis, hepatocellular carcinoma (HCC), toxic hepatitis, and others. Donor-related variables included age, sex, and BMI.

Preoperative laboratory values included white blood cell count and hemoglobin, international normalized ratio, fibrinogen, albumin, total bilirubin, sodium, and serum creatinine levels. Preoperative NLR, platelet-lymphocyte ratio (PLR), prognostic nutritional index (PNI), and C-reactive protein/albumin ratio were also collected. NLR was defined as the ratio between absolute neutrophil count versus absolute lymphocyte count, while PLR was defined as the ratio between absolute platelet count versus absolute lymphocyte count. PNI was calculated using serum albumin and total lymphocyte count [19].

Intraoperative variables included total ischemic time, post-reperfusion syndrome, graft-to-recipient weight ratio, and intraoperative transfusion requirement. Immunosuppressant-related variables included steroid, mycophenolate mofetil (MMF), cyclosporine, or tacrolimus use, and acute cellular rejection.

### 2.3. Identification of HZ and Immunosuppression

HZ and PHN were classified through a detailed chart review and database search for all subjects with relevant disease classification codes.

Immunosuppression protocols consisted of an interleukin-2 receptor inhibitor, an intraoperative steroid bolus (5–10 mg/kg), an intravenous (IV) or oral calcineurin inhibitor (CNI) such as tacrolimus or cyclosporine-based therapy, corticosteroid recycling beginning on day 1, and adjunctive MMF as previously described [20]. After the LDLT, MMF was used for patients showing tacrolimus- or cyclosporine-associated adverse effects or to augment the immunosuppression. Corticosteroids were rapidly tapered within the first 3 months. ABO-incompatible LT patients were treated with rituximab (anti-CD20 monoclonal antibody), plasmapheresis, methylprednisolone, and a prostaglandin E1 infusion [21]. Acute cellular rejection was diagnosed as previously described [22] and treated with IV methylprednisolone bolus therapy daily for 3 days.

### 2.4. Primary and Secondary Outcomes

The primary outcome was to investigate the association between preoperative NLR and HZ development. We also evaluated the association between preoperative NLR and PHN. PHN was defined as persistent chronic pain for >3 months after the diagnosis of HZ [23]. The secondary outcome was to analyze the risk factors associated with HZ and PHN. In addition, we evaluated the predictive power of NLR for HZ and PHN using the concordance index (C-index) and integrated discrimination improvement (IDI) analysis.

### 2.5. Statistical Analyses

Data are expressed as mean (standard deviation) or number (percentage) as appropriate. The data variables included in this study were compared between the HZ and non-HZ groups using the independent t-test or Mann–Whitney U-test for continuous variables or the Chi-squared or Fisher’s exact test for categorical variables. We calculated the incidence rate per 1000 person-years as the number of cases divided by 1000 person-years, that is, the incidence rate = the number of cases/sum of time spent in the study across all participants × 1000. The cumulative incidence rate of HZ was calculated using the Kaplan–Meier method. We performed Cox proportional hazard regression models with robust standard errors to estimate the hazard ratios (HRs) for HZ and PHN. All variables with *p* values < 0.1 in the univariate analysis were included in the multivariate analysis. To analyze the discriminative power of preoperative NLR for predicting HZ and PHN, a clinical model was created with the previously known risk factors (age, MMF use, sex, and HBV infection), and NLR was added to calculate the C-index and perform the IDI analysis.

In all the statistical analyses, *p* values < 0.05 were considered significant. All data were analyzed using R (version 3.1.2; R Foundation for Statistical Computing, Vienna, Austria) and IBM SPSS (version 22; IBM Corp., Armonk, NY, USA).

## 3. Results

### 3.1. Incidence of HZ

Of the 1745 patients who underwent LDLT, 57 were excluded from the study. Thus, a total of 1688 patients were included and divided into the non-HZ (*n* = 1550 [91.8%]) and HZ (*n* = 138 [8.2%]) groups (Figure 1).

**Figure 2:**
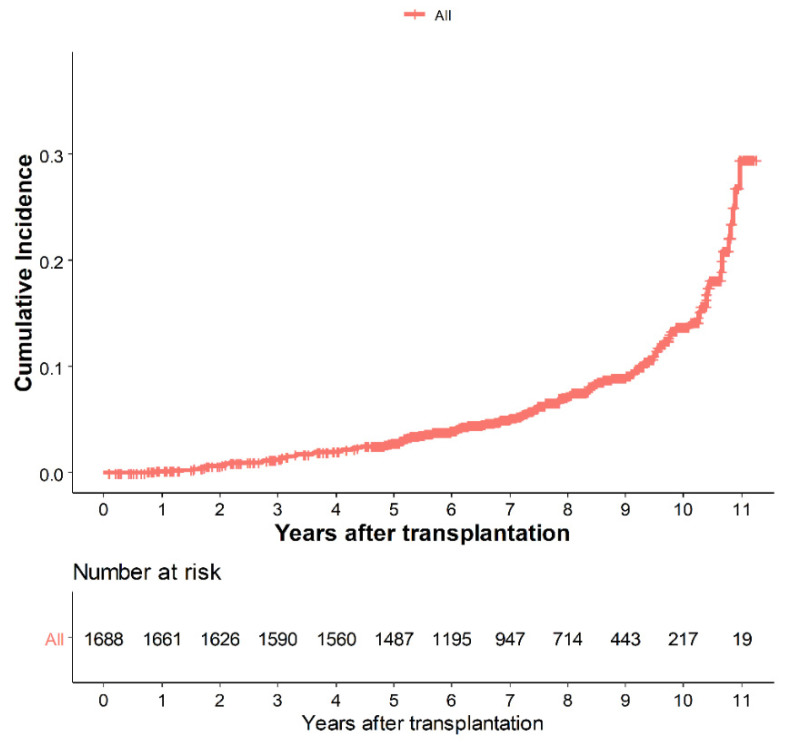
Cumulative incidence of herpes zoster infection after living donor liver transplantation. The incidence at 1, 3, 5, 8, and 10 years was 0.1%, 1.3%, 2.9%, 7.2%, and 13.5%, respectively.

Figure 2 shows the cumulative incidence of HZ according to the Kaplan–Meier method. The median follow-up time was 7.43 years (range, 2.67–10.66 years). The incidence of HZ at 1, 3, 5, 8, and 10 years was 0.1%, 1.3%, 2.9%, 7.2%, and 13.5%, respectively. The incidence of HZ was 11.2 per 1000 person-years, while that of PHN was 12.8 per 1000 person-years in 1688 patients who underwent living donor LT.

### 3.2. Patient Characteristics

Table 1 shows the patients’ baseline characteristics and perioperative variables. The mean patient age was 53.04 years, and a male predominance was evident. The mean MELD and Child–Pugh scores were 15.17 and 8.48, respectively, and 430 patients (25.5%) had a Child–Pugh class of C. Of 1705 patients, 333 (19.7%) underwent an ABO-incompatible transplantation and 10 (0.6%) required retransplantation. The etiologies included HBV (*n* = 1085 [64.3%]), HCV (*n* = 132 [7.8%]), alcoholic liver cirrhosis (*n* = 290 [17.2%]), HCC (*n* = 852 [50.5%]), toxic hepatitis (*n* = 25 [1.5%]), and others (*n* = 0 [0.1%]). The mean donor age was 27.32 years, and donors were more frequently male. The mean preoperative NLR value was 3.36. Among the CNI, tacrolimus was used in 1519 patients (90.0%), while cyclosporine was used in 169 patients (10.0%). Steroids were used in 1672 patients (99.1%), while MMF was used in 1322 patients (78.3%). Acute cellular rejection was diagnosed in 159 patients (9.4%), all of whom were treated with IV steroid pulse therapy.

The HZ group was significantly older and included more female patients. The mean preoperative NLR value and frequency of MMF use were significantly higher in the HZ group. There were no significant intergroup differences in the other study variables.

### 3.3. Primary Outcomes

In the Cox proportional hazard regression analysis, preoperative NLR was significantly associated with HZ development (HR, 1.05; 95% confidence interval [CI], 1.02–1.09; *p* = 0.005) (Table 2) and PHN development (HR, 1.08; 95% CI, 1.03–1.13; *p* = 0.001) (Table 3).

### 3.4. Secondary Outcomes

In addition, age (HR, 1.06; 95% CI, 1.04–1.09; *p* < 0.001), male sex (HR, 0.66; 95% CI, 0.46–0.95; *p* = 0.026), HBV infection (HR, 0.68; 95% CI, 0.48–0.96; *p* = 0.030), and MMF use (HR, 1.81; 95% CI, 1.11–2.97; *p* = 0.019) were significantly associated with HZ development (Table 2). In the Cox proportional hazard regression analysis of risk factors for PHN, age (HR, 1.11; 95% CI, 1.07–1.16; *p* < 0.001), and male sex (HR, 0.39; 95% CI, 0.22–0.67; *p* < 0.001) were significantly associated with PHN development (Table 3). Supplementary Figure S1A,B shows the Kaplan–Meier curves representing the incidence of HZ and PHN according to gender, respectively (log-rank test; *p* = 0.046 in HZ, *p* < 0.001 in PHN). The addition of preoperative NLR to the clinical model for predicting HZ consisting of age, MMF, sex, and HBV infection showed no significant improvement in C-index (*p* = 0.712) but showed significant discriminative power in the IDI analysis (HR, 0.008; 95% CI, 0.001–0.029; *p* = 0.020) (Table 4). The addition of preoperative NLR to the clinical model for predicting PHN consisting of age, MMF, sex, and HBV infection also showed no significant improvement in C-index (*p* = 0.523) but showed significant discriminative power in the IDI analysis (HR, 0.015; 95% CI, 0.000–0.059; *p* = 0.047) (Table 4).

## 4. Discussion

In the present study, the incidence of HZ after LDLT was 12.8 per 1000 person-years, a result that was higher than the previously reported median incidence of HZ in the general population worldwide (4–4.5 per 1000 person-years) [24]. Our study demonstrated that the preoperative NLR was strongly associated with the incidence of HZ and PHN in LDLT patients. Other risk factors for HZ were age, sex, MMF, and HBV infection. In addition, the IDI analysis showed the discriminative power of preoperative NLR. These results suggest that preoperative NLR might be an independent predictor for HZ and PHN in LDLT patients.

HZ and associated complications such as PHN may cause chronic debilitating pain, leading to poor quality of life [25,26]. HZ is also associated with a significant increase in medical expenses [27]. Despite its impact on quality of life and potential for complications, information about the risk factors associated with HZ in LDLT remains limited. Distinct from previous studies, the current study may have clinical significance, as it is the first to investigate the association between preoperative NLR and HZ in LDLT patients.

In our Cox regression analysis, preoperative NLR, age, sex, HBV infection, and MMF use were significantly associated with HZ, while NLR, age, and sex were significantly associated with PHN.

Age was reportedly an important risk factor affecting the incidence of HZ in several studies. Cell-mediated immunity generally decreases in old age [26] and may increase VZV reactivation due to a decrease in VZV-specific memory CD4 T cells [24]. In the general Korean population, the incidence rate of HZ was more than half of the population over 50 years of age, while the maximum onset age range was 60–69 years [28].

In previous general population studies, the incidence of HZ was higher in females [29]. Fleming and colleagues suggested that there is a real sex-based difference in response to the reactivation of a potential viral disease [30]. Ko and colleagues reported that female sex was the only independent risk factor for HZ after renal transplantation [31]. In a large cohort of Korean population studies, HBV infection was reportedly associated with a reduced risk of HZ due to the inhibition of VZV reactivation [32].

Some previous studies identified MMF as a risk factor for HZ after renal and heart transplantation [9,33,34]. Recent reports demonstrated that the use of MMF is an independent risk factor for HZ in LT patients [12,13]. MMF is widely used with CNI for maintenance immunosuppression to reduce the use of steroids and CNI-induced side effects such as nephrotoxicity and neurotoxicity [35,36]. MMF reportedly inhibits inosine 5′-monophosphate dehydrogenase, a key enzyme in the purine synthesis pathway in lymphocytes [37]. T and B lymphocytes produce only guanosine nucleotides through this pathway; therefore, the cellular proliferation of T and B lymphocytes is inhibited by MMF [38]. VZV-specific T-cell immunity is important to preventing HZ after a primary infection, and the MMF-induced reduction of circulating VZV-specific T lymphocytes may be associated with an increased risk of VZV reactivation [39].

Other immunosuppressants such as tacrolimus, cyclosporine, and steroids reportedly had no significant association with the incidence of HZ in LT patients [12,13], and one study reported that cyclosporine was not associated with viral infection [40]. Our results were the same. The clear mechanism of immunosuppressants for HZ development in LT patients should be studied in more detail in the future.

An elevated NLR indicates increased neutrophil and decreased lymphocyte counts: Lymphopenia reflects an impaired cell-mediated immunity, while neutrophilia is a response to systemic inflammation [41]. Neutrophil activation can trigger immunosuppression by inhibiting T-cell proliferation and cytotoxic activity via binding of PD-L1 on the surface of neutrophils to PD-1 on T cells [42]. NLR is significantly associated with circulating myeloid-derived suppressor cells, which are correlated with immune suppression and inflammation [43]. Thus, elevated NLR indicates inhibition of the proliferation of T and B lymphocytes and immune suppression, which may be associated with HZ infection. In our study, preoperative NLR was more strongly correlated in PHN patients, which strongly suggests that there is a relationship between immunosuppression degree and PHN. In a recent study, among patients with Ramsay Hunt syndrome, a complication of HZ, those with a higher NLR showed poorer outcomes, indicating that NLR is associated with HZ prognosis [44].

This study has several limitations. First, the main limitations are those inherent to retrospective studies. Thus, there is the potential for bias associated with miscoding and misclassification. Second, our data are based on the information listed in medical records collected by a single medical center, with over 99% of the patients having Korean ethnicity. Therefore, there is a possibility of results bias due to similar or homogeneous grouping, and our findings may not be generalizable to other ethnic groups. Third, our center has performed more than 300 LDLT procedures per year since 2010 [45] and our LT team has extensive experience. Therefore, our results may differ from those of studies conducted by other institutions or multicenter studies. Fourth, no studies to date have determined the preoperative NLR cutoff values that predict postoperative HZ development in LDLT recipients. Therefore, further well-designed studies are needed to validate our findings.

In conclusion, our results indicate that the preoperative NLR can provide useful clinical information about the development of HZ and PHN in LDLT recipients.

## Figures and Tables

**Table 1:** Cox regression analysis of the risk factors associated with herpes zoster.

	Study Population			
	Total (*n* = 1688)	Non-HZ (*n* = 1550)	HZ (*n* = 138)	*p*-Value
Demographic variables				
Age, years	53.04 ± 8.03	52.82 ± 8.02	55.56 ± 7.68	<0.001
Sex, male	1276 (69.68)	1180 (76.13)	96 (66.57)	0.106
BMI, kg/m^2^	24.13 ± 3.21	24.13 ± 3.16	24.21 ± 3.78	0.786
DM	410 (24.29)	374 (24.13)	36 (26.09)	0.682
HTN	274 (16.23)	247 (15.94)	27 (19.57)	0.323
CAD	118 (6.99)	111 (7.16)	7 (5.07)	0.467
MELD score	15.17 ± 8.48	15.11 ± 8.46	15.79 ± 8.62	0.369
Child–Pugh score	7.92 ± 2.19	7.90 ± 2.20	8.10 ± 2.18	0.306
Child–Pugh classification			0.473	
A	562 (33.29)	520 (33.55)	42 (30.43)	
B	696 (41.23)	641 (41.35)	55 (39.86)	
C	430 (25.47)	389 (25.10)	41 (29.71)	
ABO incompatibility	333 (19.73)	301 (19.42)	32 (23.19)	0.340
Retransplantation	10 (0.59)	9 (0.58)	1 (0.72)	0.575
Etiology				
HBV	1085 (64.28)	1003 (64.71)	82 (59.42)	0.250
HCV	132 (7.82)	121 (7.81)	11 (7.97)	0.923
Alcoholic liver cirrhosis	290 (17.18)	267 (17.23)	23 (16.67)	0.961
HCC	852 (50.47)	786 (50.71)	66 (47.83)	0.575
Toxic hepatitis	25 (1.48)	24 (1.55)	1 (0.72)	0.716
Other	1 (0.06)	1 (0.06)	0 (0.00)	1.000
Donor-related variables				
Age, years	27.32 ± 7.68	27.35 ± 7.68	26.99 ± 7.71	0.598
Sex, male	1229 (72.81)	1128 (72.77)	101 (73.19)	0.996
BMI, kg/m^2^	22.92 ± 2.91	22.89 ± 2.90	23.28 ± 3.02	0.130
Laboratory variables				
WBC	3.81 ± 2.73	3.81 ± 2.76	3.78 ± 2.47	0.899
Hemoglobin	10.79 ± 2.21	10.81 ± 2.22	10.54 ± 2.11	0.169
INR	1.48 ± 0.52	1.48 ± 0.53	1.51 ± 0.48	0.496
Fibrinogen	176.53 ± 76.70	176.98 ± 77.46	171.54 ± 67.68	0.425
Albumin	3.16 ± 0.59	3.16 ± 0.59	3.12 ± 0.55	0.437
Total bilirubin	5.15 ± 8.61	5.12 ± 8.53	5.57 ± 9.46	0.555
Sodium	137.81 ± 5.14	137.79 ± 5.17	137.99 ± 4.87	0.656
Creatinine	0.93 ± 0.85	0.93 ± 0.87	0.91 ± 0.68	0.814
NLR	3.36 ± 3.72	3.29 ± 3.60	4.13 ± 4.88	0.011
PLR	95.17 ± 67.03	95.13 ± 67.40	95.67 ± 63.05	0.927
PNI	36.07 ± 6.50	36.13 ± 6.55	35.44 ± 5.92	0.233
CRP/albumin	0.25 ± 0.52	0.24 ± 0.53	0.26 ± 0.44	0.168
Intraoperative variables				
Total ischemic time, min	127.08 ± 29.06	127.23 ± 29.26	125.44 ± 26.66	0.487
Post-reperfusion syndrome	894 (52.96)	815 (52.58)	79 (57.25)	0.335
GRWR		1.13 ± 0.24	1.12 ± 0.24	1.16 ± 0.27
Intraoperative transfusion	1359 (80.51)	1239 (79.94)	120 (86.96)	0.060
Immunosuppressant variables				
Steroid use	1672 (99.05)	1537 (99.16)	135 (97.83)	0.137
MMF use	1322 (78.32)	1203 (77.61)	119 (86.23)	0.025
Cyclosporine use	169 (10.01)	160 (10.32)	9 (6.52)	0.201
Tacrolimus use	1519 (89.99)	1390 (89.68)	129 (93.48)	0.201
Acute cellular rejection	159 (9.42)	143 (9.23)	16 (11.59)	0.447

BMI, body mass index; CAD, coronary arterial disease; CRP, C-reactive protein; DM, diabetes mellitus; GRWR, graft-to-recipient weight ratio; HBV, hepatitis B virus; HCC, hepatocellular carcinoma; HCV, hepatitis C virus; HTN, hypertension; HZ, herpes zoster; INR, international normalized ratio; MELD, Model for End-stage Liver Disease; MMF, mycophenolate mofetil; NLR, neutrophil-lymphocyte ratio; PLR, platelet-lymphocyte ratio; PNI, prognostic nutritional index; SD, standard deviation; WBC, white blood cell. Values are expressed as mean ± SD, median (interquartile range), or *n* (percentage).

**Table 2:** Cox regression analysis of the risk factors associated with herpes zoster.

	Univariate			Multivariate		
	HR	95% CI	*p*-Value	HR	95% CI	*p*-Value
NLR	1.05	1.02–1.09	0.003	1.05	1.02–1.09	0.005
Age, years	1.06	1.04–1.09	<0.001	1.06	1.04–1.09	<0.001
Sex, male	0.69	0.48–0.99	0.047	0.66	0.46–0.95	0.026
BMI, kg/m^2^	1.00	0.95–1.06	0.941			
DM	1.06	0.73–1.55	0.748			
HTN	1.35	0.89–2.06	0.160			
CAD	0.83	0.39–1.76	0.623			
MELD score	1.00	0.98–1.02	0.778			
Child–Pugh score	1.02	0.95–1.10	0.576			
Child–Pugh classification		0.753				
A						
B	1.05	0.70–1.56	0.827			
C	1.17	0.76–1.80	0.465			
HBV	0.74	0.53–1.04	0.085	0.68	0.48–0.96	0.030
HCV	1.05	0.57–1.93	0.887			
Alcoholic liver cirrhosis	1.10	0.70–1.71	0.690			
HCC	0.86	0.62–1.20	0.368			
Toxic hepatitis	0.61	0.09–4.32	0.623			
Retransplantation	1.41	0.20–9.99	0.734			
Calcineurin inhibitor						
Tacrolimus	1.00 (Ref.)			1.00 (Ref.)		
Cyclosporine	0.53	0.27–1.03	0.063	0.54	0.27–1.08	0.082
MMF	1.77	1.09–2.87	0.021	1.81	1.11–2.97	0.019
Steroid	0.68	0.22–2.14	0.515			
ACR	1.39	0.83–2.33	0.219			
GRWR	1.53	0.80–2.94	0.203			
PLR	1.00	1.00–1.00	0.536			
PNI	0.98	0.96–1.01	0.235			
CRP/albumin	1.14	0.93–1.39	0.219			
Total ischemic time, hour	1.00	0.99–1.00	0.564			
Transfusion	1.43	0.88–2.35	0.155			

CR, acute cellular rejection; BMI, body mass index; CAD, coronary arterial disease; CI, confidence interval; CRP, C-reactive protein; DM, diabetes mellitus; GRWR, graft-to-recipient weight ratio; HBV, hepatitis B virus; HCC, hepatocellular carcinoma; HCV, hepatitis C virus; HR, hazard ratio; HTN, hypertension; MELD, Model for End-stage Liver Disease; MMF, mycophenolate mofetil; NLR, neutrophil-lymphocyte ratio; PLR, platelet-lymphocyte ratio; PNI, prognostic nutritional index.

**Table 3:** Cox regression analysis of risk factors associated with postherpetic neuralgia.

	Univariate			Multivariate		
	HR	95% CI	*p*-Value	HR	95% CI	*p*-Value
NLR	1.08	1.03–1.13	<0.001	1.08	1.03–1.13	0.001
Age, years	1.11	1.07–1.16	<0.001	1.11	1.07–1.16	<0.001
Sex, male	0.40	0.23–0.69	<0.001	0.39	0.22–0.67	<0.001
BMI, kg/m^2^	0.97	0.89–1.05	0.446			
DM	1.30	0.73–2.34	0.377			
HTN	1.11	0.55–2.27	0.768			
CAD	1.53	0.61–3.83	0.366			
MELD score	1.00	0.97–1.03	0.853			
Child–Pugh score	1.02	0.91–1.15	0.692			
Child–Pugh classification		0.915				
A						
B	1.11	0.59–2.11	0.750			
C	1.15	0.57–2.33	0.689			
HBV	0.72	0.42–1.24	0.241	0.81	0.46–1.41	0.454
HCV	1.57	0.67–3.65	0.302			
Alcoholic liver cirrhosis	0.55	0.22–1.38	0.207			
HCC	0.78	0.46–1.34	0.374			
Toxic hepatitis	1.59	0.22–11.44	0.646			
Retransplantation	0.0000		0.962			
Calcineurin inhibitor						
Tacrolimus	1.00 (Ref.)					
Cyclosporine	0.48	0.15–1.52	0.213			
MMF	1.60	0.76–3.38	0.221	1.87	0.88–3.98	0.106
Steroid	0.37	0.09–1.53	0.173			
ACR	1.59	0.72–3.50	0.255			
GRWR	1.55	0.54–4.46	0.417			
PLR	1.00	1.00–1.01	0.444			
PNI	0.98	0.95–1.02	0.365			
CRP/albumin	1.12	0.81–1.55	0.500			
Total ischemic time, hour	1.00	0.99–1.00	0.327			
Transfusion	2.76	1.00–7.60	0.051	2.28	0.82–6.35	0.116

ACR, acute cellular rejection; BMI, body mass index; CAD, coronary arterial disease; CI, confidence interval; CRP, C-reactive protein; DM, diabetes mellitus; GRWR, graft-to-recipient weight ratio; HBV, hepatitis B virus; HCC, hepatocellular carcinoma; HCV, hepatitis C virus; HR, hazard ratio; HTN, hypertension; MELD, Model for End-stage Liver Disease; MMF, mycophenolate mofetil; NLR, neutrophil-lymphocyte ratio; PLR, platelet-lymphocyte ratio; PNI, prognostic nutritional index.

**Table 4:** Predictive models for herpes zoster and postherpetic neuralgia.

		C-Index (95%CI)	C-Index Difference (95% CI)	*p*-Value	IDI (95% CI)	*p*-Value
HZ	Model 1 *	0.678				
	Model 2 †	0.676	−0.002 (-−0.014–0.010)	0.712	0.008 (0.001–0.029)	0.020
PHN	Model 1 *	0.766				
	Model 2 †	0.772	0.006 (-−0.012–0.024)	0.523	0.015 (0.000–0.059)	0.047

* Model 1 = age + MMF + sex + HBV. † Model 2 = age + MMF + sex + HBV + NLRCI, confidence interval; HBV, hepatitis B virus; HZ, herpes zoster; IDI, integrated discrimination improvement; MMF, mycophenolate mofetil; NLR, neutrophil-lymphocyte ratio; PHN, postherpetic neuralgia.

## Data Availability

The dataset used and/or analyzed during the current study is available from the corresponding author upon reasonable request.

## Supplementary Materials

The following are available online at https://www.mdpi.com/article/10.3390/biomedicines9080963/s1.
