# Supplementary figures and images for: Association between Neutrophil-Lymphocyte Ratio and Herpes Zoster Infection in 1688 Living Donor Liver Transplantation Recipients at a Large Single Center
Source: Biomedicines. 2021 Aug 5;9(8):963. doi: 10.3390/biomedicines9080963 (PMC8391531; doi:10.3390/biomedicines9080963)

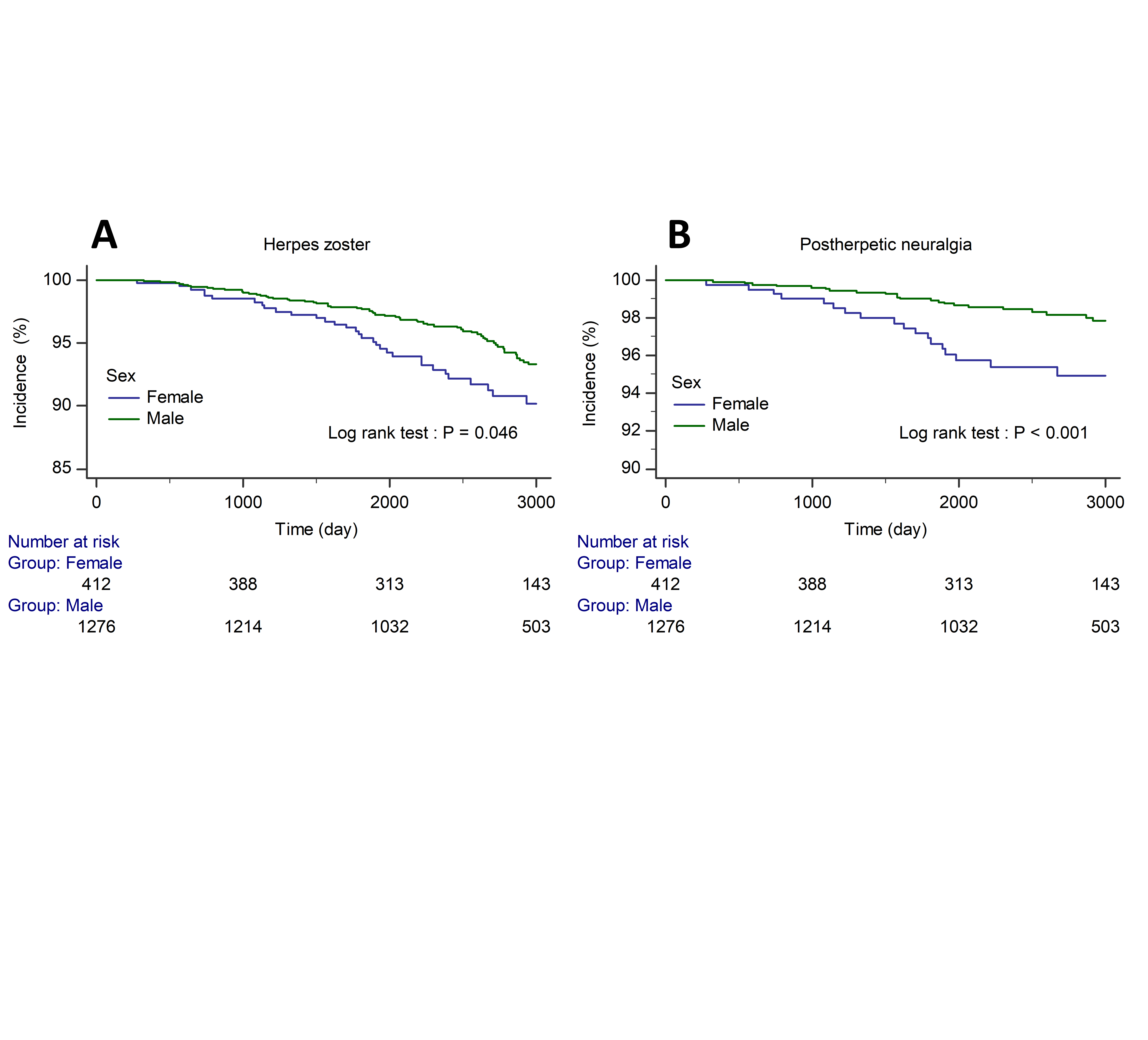

Supplement: Supplementary file 1 [file biomedicines-09-00963-s001.zip › Supplementary figure 1.tif]
